# A Follicle Size Window of Competence for In Vitro Embryo Production in High-Producing Dairy Cows: Evidence from OPU-IVP Performance and Follicular Fluid Profiling

**DOI:** 10.3390/ani16020274

**Published:** 2026-01-16

**Authors:** Mingmao Yang, Zhibing Wang, Baoli Shen, Shangnan Li, Yaochang Wei, Yifan Li, Longgang Yan, Mengkun Sun, Dong Zhou, Yaping Jin

**Affiliations:** 1 Department of Clinical Veterinary Medicine, College of Veterinary Medicine, Northwest A&F University, Yangling 712100, China; mingmao_yang@163.com (M.Y.); 18739294062@163.com (Z.W.); shenbaoli2002@126.com (B.S.); 15181873432@163.com (S.L.); 18428374393@163.com (Y.W.); yifanli18931178421@163.com (Y.L.); vetlonggangyan@163.com (L.Y.); 15667200523@163.com (M.S.); 2 Key Laboratory of Animal Biotechnology of the Ministry of Agriculture and Rural Affairs, Northwest A&F University, Yangling 712100, China

**Keywords:** follicle diameter, oocyte competence, OPU-IVP, metabolomics, dairy cow

## Abstract

A key objective of the dairy industry is to balance genetic progress with reproductive efficiency. Ovum pick-up followed by in vitro embryo production (OPU-IVP) is a pivotal technology for accelerating genetic gain. However, the relationship between follicle size and oocyte developmental competence in high-producing dairy cows under hormonal stimulation remains to be fully elucidated. This study systematically evaluated the effects of follicle diameter ovum pick-up on OPU-IVP outcomes and the underlying follicular fluid (FF) microenvironment. A total of 109 high-yielding Holstein cows were subjected to ovarian stimulation and OPU. Follicles were categorized as small (2.0–5.9 mm), medium (6.0–9.9 mm), or large (10.0–20.0 mm). Oocyte recovery, quality, and developmental competence were assessed. FF was analyzed for hormonal profiles, including anti-Müllerian hormone (AMH), estradiol (E2), follicle-stimulating hormone (FSH), and progesterone (PROG); oxidative stress markers, including malondialdehyde (MDA), glutathione peroxidase (GPx), superoxide dismutase (SOD), and total antioxidant capacity (T-AOC); and untargeted metabolomics (*n* = 10 per group). Consistently, oocytes from medium follicles exhibited superior developmental competence, achieving the highest maturation (89.93%), cleavage (72.19%), and blastocyst rates (41.88%). In contrast, large follicles had a low recovery rate (32.64%), a high proportion of degenerated oocytes (32.00%), and reduced embryonic efficiency. Metabolomic profiling revealed distinct microenvironmental differences, with medium follicles enriched in pathways like pyruvate metabolism and arachidonic acid metabolism indicating an optimal metabolic state. Hormonally, AMH decreased while E2 and PROG increased with follicle size. Large follicles exhibited significantly elevated MDA levels, indicating oxidative stress, without a concurrent rise in antioxidant capacity. In conclusion, while small follicles provide an abundant source of morphologically good oocytes, medium follicles (6.0–9.9 mm) represent a distinct “window of competence” for OPU-IVP, characterized by a follicular microenvironment most conducive to embryo production. Excessive reliance on large follicle aspiration should be avoided due to signs of over-maturity and oxidative damage. These findings provide a physiological basis for optimizing OPU strategies to enhance IVP efficiency in high-producing dairy cows.

## Simple Summary

Ovum pick-up followed by in vitro embryo production (OPU-IVP) is an important technology for accelerating genetic gain in dairy cattle. In high-producing cows subjected to hormonal stimulation, however, the association between follicle size and oocyte developmental competence remains equivocal. Here, 109 high-yielding Holstein cows were used to determine how follicular diameter—small (2.0–5.9 mm), medium (6.0–9.9 mm), and large (10.0–20.0 mm)—affects OPU-IVP performance and to investigate potential underlying mechanisms by integrating follicular fluid (FF) hormone profiles, oxidative stress indices, and untargeted metabolomics. Small follicles yielded the greatest number of oocytes and the highest proportion of morphologically normal oocytes. Nevertheless, oocytes recovered from medium-sized follicles exhibited superior developmental competence, with the highest rates of maturation, fertilization, and progression to transferable embryos, achieving an overall blastocyst rate of approximately 41.88% compared with 29.11% for oocytes from small follicles. In contrast, large follicles produced fewer oocytes, showed a higher proportion of degenerated oocytes, and were associated with increased lipid peroxidation. Collectively, these results identify medium-sized follicles as the optimal developmental window for oocyte retrieval in high-producing dairy cows. Emphasizing collection within this follicle size range may enhance embryo yield, minimize procedural losses, and improve the efficiency and sustainability of breeding programs.

## 1. Introduction

A key objective in the dairy industry is to balance genetic progress with reproductive efficiency [1]. Among assisted reproductive biotechnologies, ovum pick-up followed by in vitro embryo production (OPU-IVP) is regarded as an important strategy to accelerate genetic improvement through the maternal line [2,3]. To increase the efficiency of oocyte recovery, ovarian stimulation is often applied before oocyte collection to synchronize follicular wave development [4,5], and transvaginal ultrasonography is used to assess follicular morphology [6]; however, such stimulation protocols may exacerbate developmental heterogeneity within the follicular cohort and complicate the selection of follicles and oocytes. Follicular diameter is one of the key determinants of oocyte developmental competence, and follicle size is closely associated with oocyte quality, maturity, fertilizability, and embryo developmental potential [7,8,9].

Nevertheless, findings on the effect of follicle size on oocyte competence remain inconsistent. Some reports indicate that small follicles (≤4 mm) yield higher oocyte recovery rates but limited developmental potential [10], whereas oocytes collected from medium to large follicles exhibit greater developmental capacity [5,11]; other studies have reported no differences among follicle size categories [2,12]. In addition, several studies suggest that oocytes retrieved from follicles in the growing phase of the follicular wave or the early dominance phase have superior competence [13,14]. It should be noted that part of the existing evidence derives from abattoir-sourced ovaries, which raises concerns about applicability to live, managed cattle [10,15]. Collectively, these discrepancies underscore that the “optimal” follicle size may be species- and protocol-specific.

Follicular fluid (FF), which constitutes the immediate microenvironment of the oocyte, plays a pivotal role in regulating oocyte developmental competence, maturation quality, and subsequent embryogenesis. It provides crucial nutritional support and growth factors by accumulating diverse metabolites, including glucose, amino acids, fatty acids, steroid hormones, and antioxidants [16,17,18]. The metabolomic profile of FF is significantly influenced by follicle size, health status, and maternal factors such as age, nutrition, and hormonal levels [19,20]. For instance, FF from larger follicles is often characterized by higher concentrations of glucose and β-hydroxybutyrate, which may influence the quality and metabolic competence of both oocytes and granulosa cells [21]. Furthermore, the balance between antioxidants (e.g., glutathione, SOD) and oxidative stress markers (e.g., reactive oxygen species, ROS) in FF directly affects meiotic resumption, cytoplasmic maturation, and embryonic genome activation [22]. Elevated ROS levels can impair mitochondrial function and induce DNA fragmentation in oocytes, whereas enhanced antioxidant capacity in FF can mitigate oxidative damage, potentially through the regulation of the Nrf2/ARE signaling pathway, thereby improving subsequent embryo quality [23].

Although previous studies have linked follicle size with oocyte developmental competence, high-producing dairy cows experience sustained metabolic stress during lactation, which may alter the follicular microenvironment and oocyte quality [24]. Therefore, direct extrapolation of earlier findings to OPU practice in this specific population should be made cautiously and may involve uncertainty. In this context, the present study aimed to evaluate how follicular diameter influences OPU-IVP efficiency in high-producing dairy cows by examining follicular developmental capacity, metabolic pathways, and the intra-follicular hormonal and oxidative-stress milieu. Our objectives were to: (1) compare oocyte recovery rate, quality grade, maturation rate, cleavage rate, and blastocyst yield among small (2.0–5.9 mm), medium (6.0–9.9 mm), and large (10.0–20.0 mm) follicles; (2) analyze concentrations of key hormones [anti-Müllerian hormone (AMH), estradiol (E2), follicle-stimulating hormone (FSH), and progesterone (PROG)] and oxidative-stress markers [malondialdehyde (MDA), glutathione peroxidase (GPx), superoxide dismutase (SOD), and total antioxidant capacity (T-AOC)] in FF; (3) integrate metabolomics to reveal potential mechanisms underlying differences in developmental competence across follicle diameters. The anticipated results will provide a theoretical basis and practical guidance to optimize OPU strategies and improve IVP efficiency in high-producing dairy cows.

## 2. Materials and Methods

### 2.1. Farm and Animals

A total of 120 clinically healthy Holstein donor cows were selected based on genetic merit and high production performance. Eligible cows had more than three parities (5–7 years of age) and a previous 305 d milk yield ranking within the top 10% of the herd, defined as a first-lactation 305 d milk yield greater than 12,500 kg, with successive lactations producing more milk than the preceding parity. All donors were housed in a free-stall barn and fed a total mixed ration (TMR) consisting of corn silage, alfalfa hay, oat hay, corn grain, soybean meal, and cottonseed meal. The TMR was delivered three times daily, with ad libitum access to fresh water. Herd management followed standardized procedures, and housing facilities, diet formulation, and management personnel remained consistent throughout the study period. All procedures involving animals were reviewed and approved by the Animal Welfare and Ethics Committee of Northwest A&F University (Approval No. 2021028).

### 2.2. Experimental Design and Ovarian Stimulation

**Figure 1:**
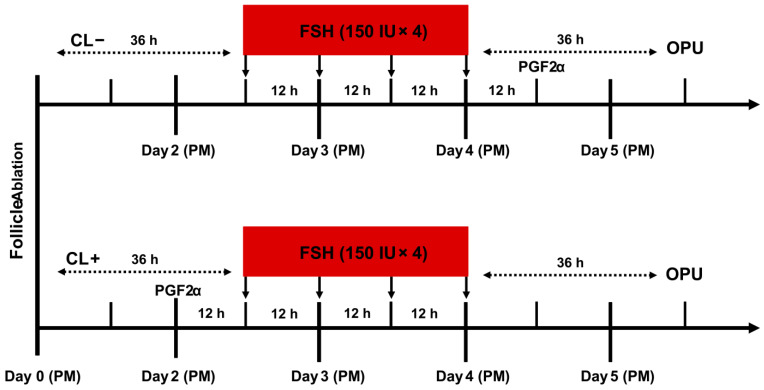
Ovarian stimulation protocol schematic.

Before the trial, donor cows were screened for ovarian status by rectal palpation and a veterinary B-mode ultrasound scanner (Easi-Scan; IMV Technologies, L’Aigle, France). Cows with static ovaries, pregnancy, or cystic follicles were excluded. Follicular wave synchronization was performed as previously described [3]. On a random day of the estrous cycle (d 0, afternoon), follicles > 5 mm were ablated under transvaginal ultrasound guidance using a portable scanner (Exapad-Mini; IMV Technologies, L’Aigle, France) to synchronize emergence of a new follicular wave. Ovarian stimulation proceeded as follows (Figure 1). If a corpus luteum (CL) was present, 25 mg prostaglandin F2α (PGF2α; Sansheng Biological Technology, Ningbo, China) was administered intramuscularly 24 h later (Day 1) to induce luteolysis. The first FSH injection (Sansheng Biological Technology, Ningbo, China) was administered 12 h after PGF2α treatment (Day 1.5), followed by four additional intramuscular injections of 150 IU FSH at 12 h intervals. Oocyte retrieval was performed 36 h after the last FSH injection (Day 5). For cows without a CL, FSH treatment (4 × 150 IU at 12 h intervals) was initiated 36 h after follicular ablation (Day 1.5). On Day 5, 25 mg PGF2α was administered intramuscularly 12 h after the final FSH injection, and OPU was performed 24 h later. Each cow was included for only one OPU session.

### 2.3. Follicle Classification and OPU

Prior to OPU, 5 mL of 2% lidocaine HCl was administered via tail epidural anesthesia; adequacy was judged by reduced tail tone. After rectal evacuation and thorough cleaning of the vulva and perineum, a portable ultrasound probe was inserted into the vagina, and the ovaries were manipulated per rectum to obtain clear images on the monitor. Follicular diameter was measured in real time using the scanner’s built-in calipers. All visible follicles > 2 mm were aspirated and categorized as small (2.0–5.9 mm), medium (6.0–9.9 mm), or large (10.0–20.0 mm); cystic follicles > 20 mm were excluded. Aspiration used a disposable OPU needle (1.20 mm × 75 mm, 18 g; 2207, Misawa Medical Industry Co., Ltd., Kasama, Japan) connected to a vacuum pump (FV 6, Fujihira Industry Co., Ltd., Tokyo, Japan) set to 70 mmHg (or a flow of 20–30 mL/min) and prewarmed flushing medium [DPBS containing 1% FCS and heparin (5000 IU/mL)] at 38.5 °C. Each follicle was aspirated individually. FF from follicles of the same size category within the same ovary and cow was pooled. After aspiration, the needle was flushed with medium to recover any cumulus–oocyte complexes (COCs) retained within the lumen. Collected FF was transported to a nearby laboratory within 20 min at a constant temperature. All FF collection and scanning were performed by two trained veterinarians using a standardized protocol.

### 2.4. COC Recovery and Grading

COCs were recovered and evaluated under a stereomicroscope (SZX7; Olympus, Tokyo, Japan) at 30× magnification. Recovered oocytes were washed 2–3 times in PBS and classified morphologically based on cytoplasmic appearance and cumulus cell distribution, with minor modifications from a previously described system [25]. This morphological grading reflects cumulus investment and ooplasm appearance and does not represent oocyte nuclear maturational/developmental stage. Grade A COCs exhibited compact, multilayered cumulus investment and homogeneous ooplasm; their overall appearance was light and translucent. Grade B COCs also possessed compact, multilayered cumulus and homogeneous cytoplasm but had a slightly rougher appearance with a darker band surrounding the oocyte; their overall color was slightly darker and less translucent. Grade C COCs had less compact cumulus investment and irregular ooplasm containing dark clusters; their overall color was darker than Grades A and B. Grade D COCs displayed expanded cumulus distribution with dark cumulus cell clumps embedded in a jelly-like matrix and irregular ooplasm with dark masses; their overall appearance was dark and irregular. In this study, completely denuded (naked) oocytes were considered non-viable and discarded, whereas all remaining COCs, including partially denuded COCs with residual cumulus layers, were cultured in vitro.

### 2.5. In Vitro Embryo Production and Evaluation

Viable COCs were pre-equilibrated by 3–5 rinses in HEPES-buffered handling medium and transferred into BO IVM medium droplets (71001; IVF Bioscience, Falmouth, UK) under mineral oil for in vitro maturation at 38.5 °C in a tri-gas incubator for 22–24 h. After maturation, COCs were transferred to BO IVF medium (71004; IVF Bioscience) and co-incubated for 12 h with frozen–thawed bull sperm. Semen straws were thawed in a 37 °C water bath for 40 s, and post-thaw sperm quality was evaluated immediately; total motility was 55.34% and progressive motility was 38.20%. Motile spermatozoa were then purified using a Percoll density gradient (40%/80%; PS40 100 and PS80 100; Nidacon, Sweden, Mölndal, Sweden), washed, and resuspended in BO IVF medium. The final sperm concentration in fertilization droplets was adjusted to 2 × 10^6^ sperm/mL. Following fertilization, cumulus cells were removed by gentle pipetting, and presumptive zygotes were cultured in BO IVC medium (71005; IVF Bioscience) for 7 d. All media and droplets were prepared in advance, pre-warmed, and covered with mineral oil. Cleavage was recorded on day 3 (number cleaved/COCs cultured), and blastocyst development was recorded on day 7 (number of blastocysts/COCs cultured).

### 2.6. FF Sample Collection and Measurement of Oxidative Stress Markers and Hormones

During OPU, FF from the different follicle size categories was collected. Before flushing the OPU system, original FF was aspirated from 2–3 follicles in each diameter category using a syringe; this fluid was collected directly from the follicle prior to flushing to avoid dilution by the flushing medium and interference from blood contamination. Only clear, non-hemolyzed samples were analyzed, and cloudy or blood-tinged samples were excluded. Samples were centrifuged (1500× *g*, 10 min), and supernatants were stored at −20 °C until measurement. Oxidative stress markers—including MDA (BC0025, Solaibao, Beijing, China), T AOC (S0121), SOD (S0101S), and GSH Px (S0059S) (Beyotime Biotechnology, Nantong, China)—were measured using commercial kits according to manufacturers’ instructions. FF hormone concentrations were determined using bovine ELISA kits (Yuanju Biotechnology, Shanghai, China) for E2 (YJ33035), PROG (YJ32780), FSH (YJ33029), and AMH (YJ33145). Briefly, pretreated FF and standards were added to 96 well plates, followed by HRP labeled detection antibodies and incubation for 60 min at 37 °C. Plates were washed five times, TMB substrate was added for 15 min in the dark, reactions were stopped, and absorbance was read at 450 nm. Standard curves were fitted with a 4-parameter logistic model and sample concentrations were corrected for dilution. Assay sensitivities were E2 < 1.0 pg/mL, FSH < 0.1 mIU/mL, AMH < 10 pg/mL, and PROG < 10 pmol/L. Intra and interassay coefficients of variation were <15%, confirming stability and repeatability.

### 2.7. Untargeted Metabolomics Analysis

Thirty FF samples (*n* = 10 per follicle size category: small, 2.0–5.9 mm; medium, 6.0–9.9 mm; large, 10.0–20.0 mm) were analyzed by Genedenovo Biotechnology Co., Ltd. (Guangzhou, China). Untargeted metabolomic profiling was performed using ultra-high-performance liquid chromatography coupled to tandem mass spectrometry (UHPLC–MS/MS). FF metabolites were extracted with prechilled methanol/acetonitrile/water (2:2:1, vol/vol/vol). Chromatographic separation was carried out on a Waters ACQUITY UPLC BEH Amide column (1.7 µm, 2.1 mm i.d. × 100 mm length) using acetonitrile and an aqueous solution of 25 mM ammonium acetate/ammonium hydroxide as the mobile phases under a gradient elution program. Mass spectra were acquired on an AB TripleTOF 6600 system operated in both positive and negative electrospray ionization modes over an *m*/*z* range of 60–1000 Da. Pooled quality control (QC) samples were injected periodically throughout the sequence to monitor instrument stability.

### 2.8. Statistical Analysis

Of the initial 120 cows, 11 were excluded from OPU due to conditions such as uterine inflammation or ketosis; therefore, data from 109 cows were included in the final analysis. The following endpoints were assessed per cow: the numbers of small (2–6 mm), medium (6–10 mm), and large (10.0–20.0 mm) follicles aspirated; total oocytes recovered; number and proportion of oocytes graded A, B, C, or D; recovery rate (total oocytes/total follicles); cleavage rate (cleaved embryos/cultured COCs); blastocyst yield (blastocysts/cultured COCs).

All statistical analyses were conducted using SAS 9.4 (SAS Institute Inc., Cary, NC, USA). Results are expressed as least squares means (LSM) with 95% confidence intervals (CI). Proportional data were analyzed using generalized linear mixed models (GLMM) with treatment group as a fixed effect and cow as a random effect. Count data were modeled with a negative binomial GLMM to account for overdispersion. Continuous outcomes were evaluated using linear mixed models (LMM). Pairwise comparisons were adjusted via the Holm method, with significance set at *p* < 0.05. For hormone and oxidative stress measures, normality and homoscedasticity were tested; where assumptions were violated, the Kruskal–Wallis H test was applied, followed by Dunn’s post hoc test with Bonferroni correction. Graphs were generated using GraphPad Prism 8.0 (GraphPad Software Inc., La Jolla, CA, USA), and significant differences between groups are indicated by different superscript letters (a, b, c).

For metabolomic data, principal component analysis (PCA) was performed in R to assess sample clustering and the reproducibility of quality control (QC) samples. For pairwise comparisons between follicle size groups, partial least squares discriminant analysis (PLS-DA) and orthogonal PLS-DA (OPLS-DA) models were constructed using the ropls package, and model performance was evaluated by cross-validation and 200-fold permutation testing. Differentially expressed metabolites (DEMs) between groups were defined as those with a variable importance in projection (VIP) value ≥ 1.0 in the OPLS-DA models and a Student’s *t*-test *p* < 0.05. Hierarchical clustering, correlation heatmaps, and volcano plots were generated in R, and KEGG-based pathway annotation together with metabolite set enrichment analysis was used to identify significantly enriched metabolic pathways.

## 3. Results

### 3.1. Oocyte Recovery and Quality Grade Distribution

A total of 1136 small follicles (2.0–5.9 mm), 1009 medium follicles (6.0–9.9 mm), and 383 large follicles (10.0–20.0 mm) were aspirated from 109 high-yielding dairy cows. The mean numbers of small and medium follicles per cow were similar [10.42 (95% CI: 9.18–11.83) vs. 9.26 (95% CI: 8.09–10.59)], and both were significantly higher than the mean number of large follicles per cow [3.51 (95% CI: 2.91–4.25); *p* < 0.05]. Follicle diameter was negatively associated with both oocyte recovery efficiency and morphological quality. As follicular diameter increased, the oocyte recovery rate decreased: 55.11% (51.85–58.32) for small follicles, 46.68% (44.23–49.15) for medium follicles, and 32.64% (29.39–36.06) for large follicles (*p* < 0.05). Likewise, the proportion of high-quality oocytes (Grade A + B) decreased with follicle size, being highest in the small-follicle group [64.22% (60.06–68.18)] and significantly greater than in the medium [48.62% (43.29–53.98)] and large groups [26.40% (18.68–35.90)]. Conversely, the proportion of degenerated oocytes (Grade D) increased with follicle size, reaching 32.00% (22.26–43.61) in large follicles compared with 19.96% (15.52–25.28) in medium follicles and 12.94% (10.21–16.27) in small follicles. The proportion of recovered COCs that were subsequently cultured (Cultured COCs/Total COCs) did not differ among groups (small: 88.01%, medium: 86.28%, large: 75.89%). Together, these results indicate that under ovarian stimulation, small follicles provide the largest number of morphologically high-quality COCs, whereas large follicles are associated with reduced recovery efficiency and a higher degeneration rate (Table 1). For transparency, pooled raw counts (n/N) are provided in Supplementary Table S1.

### 3.2. Developmental Competence of Oocytes from Different Follicle Sizes

The developmental competence of oocytes from different follicle sizes is summarized in Table 2. The number of mature oocytes per cow was similar for small and medium follicles [4.38 (3.83–5.02) and 4.32 (3.78–4.93), respectively], and both were significantly greater (*p* < 0.05) than that from large follicles [1.37 (1.16–1.61)]. A comparable pattern was observed for the number of cleaved embryos and blastocysts, with small and medium follicles yielding similar values [cleaved: 3.46 (2.94–4.08) vs. 3.56 (3.07–4.12); blastocysts: 1.63 (1.33–1.99) vs. 2.01 (1.67–2.43)], which were significantly higher than those from large follicles [cleaved: 1.24 (1.02–1.52); blastocysts: 0.59 (0.42–0.83)].

In contrast, the maturation rate was highest for medium follicles [89.93% (87.37–92.02)], significantly exceeding the rates for both small [78.48% (73.68–82.61)] and large [71.28% (62.88–78.43)] follicles. The cleavage rate was significantly higher in medium [72.19% (67.83–76.55)] and large [63.44% (52.80–74.07)] follicles compared to small follicles [57.13% (51.64–62.63)]. Notably, the blastocyst rate was greatest for oocytes derived from medium follicles [41.88% (37.51–46.37)], being significantly higher than that from small follicles [29.11% (25.19–33.38)], while the rate for large follicles [26.31 (17.91–35.79)] was intermediate and not statistically different from the small group.

### 3.3. Hormonal Profiles in FF

**Figure 2:**
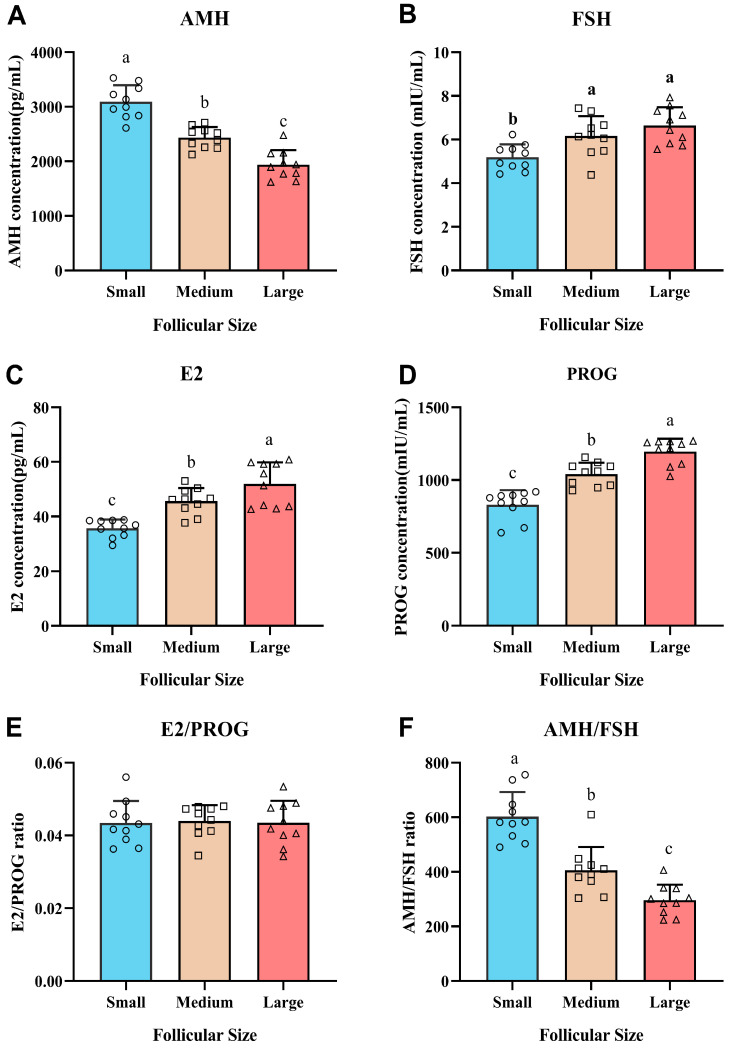
Hormonal profiles in FF aspirated from small (2.0–5.9 mm), medium (6.0–9.9 mm), and large follicles (10.0–20.0 mm) during OPU (*n* = 10 per group). (**A**) Concentrations of AMH (pg/mL), (**B**) Concentrations of FSH (mIU/mL), (**C**) Concentrations of E2 (pg/mL), and (**D**) Concentrations of PROG (ng/mL). (**E**) Ratio of E2/PROG. (**F**) Ratio of AMH/FSH. Values are presented as mean ± SD. Within each panel, bars with different lowercase letters (a, b, c) differ significantly (*p* < 0.05). Circles, squares, and triangles indicate individual samples from small, medium, and large follicles, respectively.

AMH concentration was significantly influenced by follicle diameter, with the highest level detected in small follicles, gradually decreasing in medium and large follicles (Figure 2A). In contrast, E2 concentration was positively correlated with follicle size, being lowest in small follicles and highest in large follicles (Figure 2B). FSH levels were slightly elevated in large compared to small and medium follicles (Figure 2C). As expected, PROG concentration was markedly higher in large follicles (Figure 2D). Notably, despite the significant individual changes in E_2_ and PROG concentrations, the ratio of E_2_ to PROG did not differ significantly among small, medium, and large follicles (*p* > 0.05, Figure 2E). The AMH/FSH ratio, reflecting follicular sensitivity and developmental potential, was significantly elevated in small follicle and decreased in large follicles (Figure 2F).

### 3.4. Oxidative and Antioxidant Levels in FF

**Figure 3:**
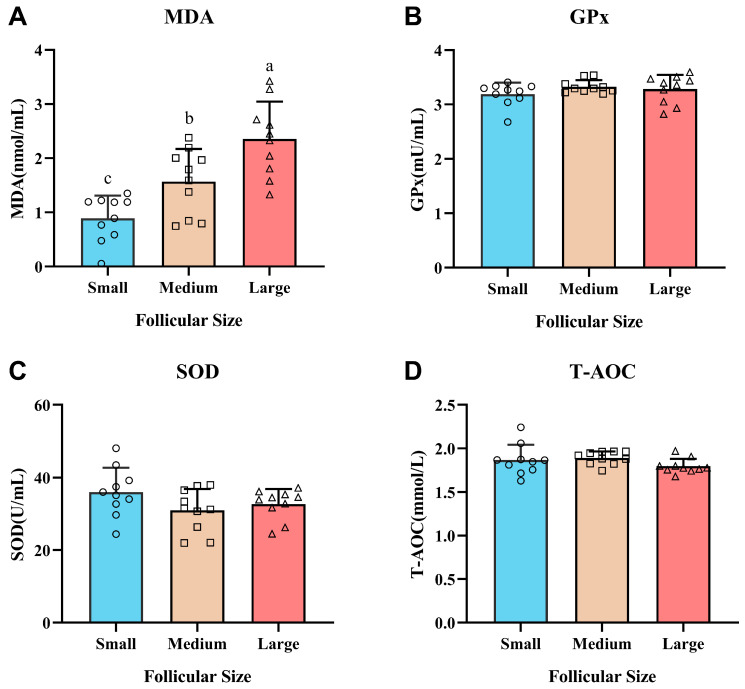
Antioxidant profiles and oxidative stress markers in FF from small (2.0–5.9 mm), medium (6.0–9.9 mm), and large follicles (10.0–20.0 mm) of high-yielding dairy cows (*n* = 10 per group). (**A**) The levels of MDA (nmol/L) in FF, (**B**) The levels of GPx (U/mL) in FF, (**C**) The levels of SOD (U/mL) in FF, (**D**) The levels of T-AOC (mmol/L) in FF. Values are presented as mean ± SD. Within a panel, bars with different lowercase letters (a, b, c) denote significant differences (*p* < 0.05). Circles, squares, and triangles indicate individual samples from small, medium, and large follicles, respectively.

As shown in Figure 2, MDA concentration was significantly affected by follicle size. It was lowest in small follicles, intermediate in medium follicles, and highest in large follicles (*p* < 0.05), indicating a progressive increase in lipid peroxidation and oxidative stress during follicular development (Figure 3A). In contrast, the activities of the key antioxidant enzymes GPx (Figure 3B) and SOD (Figure 3C), as well as the total T-AOC (Figure 3D), did not change significantly (*p* > 0.05) among small, medium, and large follicles. These results indicate that while enzymatic antioxidant defenses and total antioxidant capacity remain stable during follicular growth, large follicles exhibit a pronounced state of oxidative stress, as evidenced by elevated MDA levels.

### 3.5. Metabolic Differences in the FF from Small, Medium and Large Follicles

**Figure 4:**
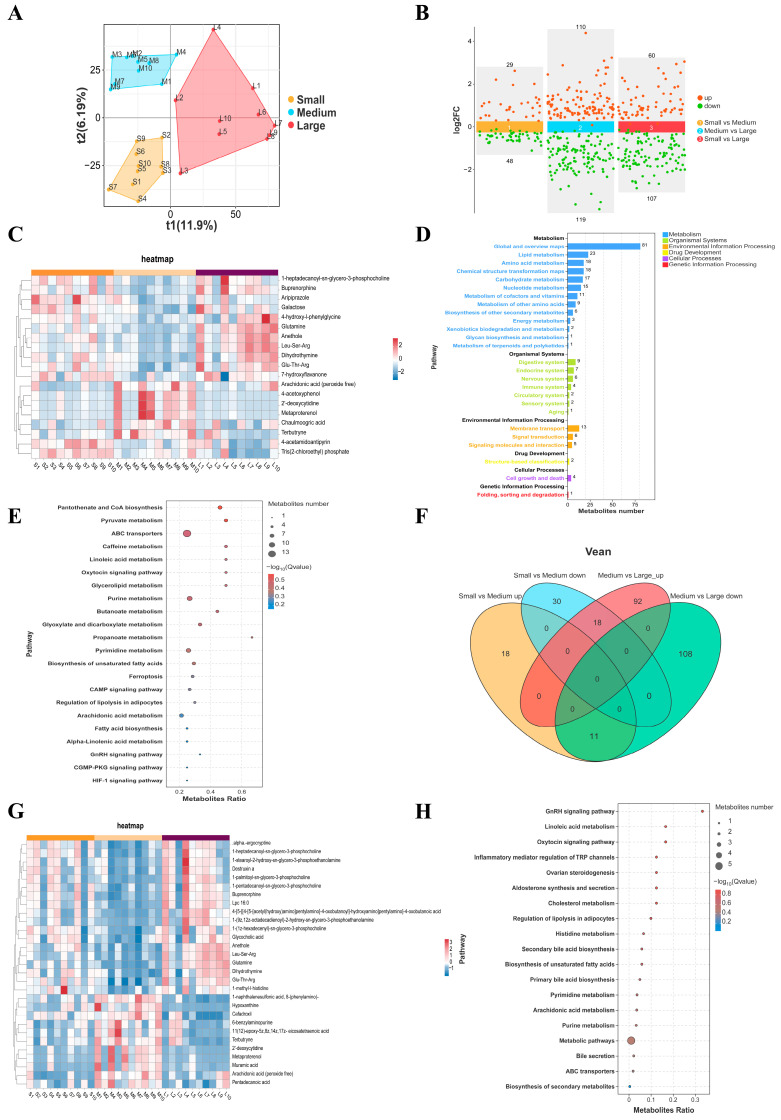
Non-targeted metabolomics analysis of FF from small, medium, and large follicles. (**A**) Partial least squares-discriminant analysis (PLS-DA) score plot showing the metabolic profile separation among the three groups. (**B**) Scatter plot of the differentially expressed metabolites (DEMs) identified across the comparisons. (**C**) Heatmap displaying the relative abundance of the top DEMs across all samples from the three groups. (**D**) Bar chart of KEGG pathway classification for the identified DEMs. (**E**) Bubble plot of KEGG pathway enrichment analysis for the DEMs. The bubble size and color represent the number of metabolites and the statistical significance, respectively. (**F**) Venn diagram illustrating the number of unique and common DEMs between the small vs. medium and medium vs. large follicle comparisons. (**G**) Heatmap of the DEMs common to both the small vs. medium and medium vs. large comparisons. (**H**) Bubble plot of KEGG pathway enrichment analysis for the common DEMs shown in (**G**).

Non-targeted metabolomics analysis was conducted on FF in the small follicle group (*n* = 10), medium follicle group (*n* = 10), and large follicle group (*n* = 10). A total of 1774 known metabolites were identified in the positive ion mode and 1309 known metabolites were identified in the negative ion mode. The results of partial least squares discriminant analysis (PLS-DA) showed that there were significant distinctions in the metabolic characteristics of the three groups of samples (Figure 4A). Based on the criteria of variable projected importance (VIP) ≥ 1 and *t*-test *p* < 0.05, DEMs between groups were screened. A total of 77 DEMs (29 up-regulated and 48 down-regulated) were identified between the small and medium follicle groups. The largest metabolic difference was observed between the medium and large follicle groups, with 229 DEMs (110 up-regulated and 119 down-regulated). In comparison, 167 DEMs (60 up-regulated and 107 down-regulated) were identified between the small and large follicle groups (Figure 4B). After normalizing all the differential metabolites among the three groups, cluster analysis was conducted and the heat maps of the first 20 DEMs were drawn (Figure 4C). The results showed that the expression levels of some metabolites, such as Arachidonic acid, 2′-deoxycytidine, Muramic acid, etc., in the medium follicle group were significantly higher than those in the small follicle group and the large follicle group. And Anethole, Leu-Ser-Arg, Dihydrothymine, Glutamine was elevated in the large follicle group. KEGG pathway enrichment analysis indicated that these differential metabolites were mainly involved in pathways such as pantothenic acid and coenzyme A biosynthesis, pyruvate metabolism, ABC transporter, oxytocin signaling pathway, and purine metabolism (Figure 4E). The Venn diagram illustrated the overlapping and unique DEMs across comparisons (small vs. medium, medium vs. large), revealing 29 common DEMs shared among the three groups (Figure 4F). These shared metabolites mainly include Arachidonic acid (peroxide free), Glycocholic acid, 2′-deoxycytidine, Hypoxanthine, Anethole, Glycocholic acid, etc. (Figure 4G). These common DEMs were significantly enriched in the GnRH signaling pathway, linoleic acid metabolism, oxytocin signaling pathway, and Biosynthesis of unsaturated fatty acids in KEGG enrichment analysis (Figure 4H).

## 4. Discussion

The present study systematically investigated the interrelationships among follicle diameter, the follicular microenvironment, and oocyte developmental competence in high-yield dairy cows. The core findings demonstrate that medium-sized follicles (6.0–9.9 mm) represent a distinct “window of competence” for OPU-IVP, characterized by a FF endocrine and metabolic profile most conducive to in vitro maturation and early embryonic development. In contrast, while small follicles yield a higher number of recoverable oocytes, their maturation, cleavage, and blastocyst rates are suboptimal. Conversely, large follicles exhibit endocrine and oxidative stress profiles indicative of over-maturity, associated with reduced embryonic efficiency.

During the initial phase of ovarian stimulation, 70–80% of small follicles (<5 mm) are responsive to exogenous gonadotropins [2,26], which may contribute to follicular size asynchrony. Modern ultrasonography enables the identification of follicles as small as 2 mm, and the number of visible antral follicles largely determines the potential COCs yield [27,28]. Mechanically, the lower volume, lower viscosity, and reduced intrafollicular pressure of small follicles contribute to decreased aspiration loss and enhanced oocyte recovery. This aligns with reports by Seneda et al., who noted significantly higher recovery rates from follicles ≤4 mm [10]. Conversely, aspirates from large follicles (>10 mm) are often more viscous, and the aspirate may contain sheets of granulosa cells, increasing the risk of COC damage or degeneration during retrieval and thereby reducing both recovery rates and morphological quality [28,29]. This pattern is consistent with our findings: small follicles (2.0–5.9 mm) exhibited the highest recovery rate (55.11%) and the highest proportion of top-quality oocytes (Grades A + B, 64.22%), whereas large follicles (10.0–20.0 mm) showed a marked decline in recovery (32.64%) and a significant increase in degenerated oocytes (Grade D, 32.00%).

Oocyte developmental competence is intrinsically linked to follicular growth [30,31]. Our developmental analysis revealed that oocytes from medium follicles achieved superior maturation (89.93%), cleavage (72.19%), and blastocyst rates (41.88%) compared to those from small and large follicles. Although the blastocyst yield per cow was similar between small (1.63) and medium (2.01) follicles, the lower maturation rate (78.48%) in the small follicle group highlights a developmental limitation. These findings collectively suggest that medium follicles represent an optimal developmental stage, wherein the FF microenvironment effectively supports both meiotic completion and early embryogenesis. Consistent with previous reports, most oocytes from small follicles (>2 mm) retain the capacity to support fertilization and embryo development [32,33]. Studies by Luciano’s and Sirard’s groups have shown that oocytes from early antral follicles (0.5–2 mm in diameter) are predominantly at the GV0 stage and exhibit a limited ability to resume meiosis [34,35]. In contrast, follicles larger than 2 mm contain oocytes distributed across GV1, GV2, and GV3 stages in roughly similar proportions, with GV2 and GV3 oocytes demonstrating greater developmental competence and a higher likelihood of developing to the blastocyst stage. However, the relationship between follicle size and oocyte competence is not linear, peaking when follicles are approximately 6–10 mm in diameter [36,37]. Follicles that are smaller (<4–5 mm) or larger (>10 mm) than this optimal range typically yield oocytes with a reduced capacity to form blastocysts [11,38]. Previous studies reported the highest blastocyst rates for bovine medium-sized follicles (6–10 mm) and showed that p-FSH super-stimulation increases the proportion of medium follicles, thereby enhancing embryo yield [5].

The hormonal profile within FF serves as a key indicator of oocyte health and maturational status [39,40,41]. AMH, primarily secreted by granulosa cells of small preantral/antral follicles, declines with follicular growth [42,43]. In ruminant studies, small follicles exhibit higher AMH concentrations compared to large ones, according to the fact that granulosa cells reduce their AMH production during final follicular maturation. This decline allows follicles to escape inhibitory regulation, facilitating selection and maturation [44,45,46]. FSH is the primary hormone responsible for inducing the growth of antral follicles during follicular waves [47]. The pre-wave rise in endogenous FSH concentration drives follicular recruitment and growth, while the subsequent decline to basal levels is necessary for the selection of a single dominant follicle [5,48]. Our study observed a progressive decrease in FF AMH and the AMH/FSH ratio with increasing follicle size, indicating active recruitment in small follicles and a shift toward selection/luteinization in medium and large follicles. PROG plays an important role in promoting oocyte maturation, ovulation, and early embryonic development, and its concentration typically increases markedly approximately 2 h before ovulation [49]; however, recent studies suggest that FF PROG concentration is not directly associated with embryo developmental competence but is more closely related to follicle size [50], and has been reported to be negatively correlated with oocyte yield and follicular antioxidant capacity [51]. A similar trend was observed in our study: both PROG and MDA concentrations were elevated in large follicles, indicating a more oxidative intrafollicular microenvironment. This pattern may also reflect the onset of early granulosa-cell luteinization in larger follicles, which could compromise oocyte developmental competence [52]. The elevated AMH/FSH ratio in small follicles further underscores their heightened sensitivity to FSH, favoring recruitment and selection [53].

Metabolomic profiling further supported the microenvironmental differences observed among follicle size categories in the present study. Consistent with the superior IVP performance of medium follicles, follicular fluid (FF) from medium follicles showed significant upregulation of metabolites such as arachidonic acid, 2′-deoxycytidine, and muramic acid. Arachidonic acid, a key polyunsaturated fatty acid, is a precursor for prostaglandins and leukotrienes and has been implicated in cumulus expansion and oocyte maturation [54,55]. In our dataset, medium follicles achieved the highest maturation rate (89.93%), cleavage rate (72.19%), and blastocyst rate (41.88%), suggesting that the enrichment of arachidonic acid-related signaling may reflect a balanced microenvironment that supports oocyte–cumulus communication, membrane dynamics, and appropriate inflammatory tone. Likewise, 2′-deoxycytidine, a precursor for DNA synthesis, may indicate active cellular proliferation and repair in granulosa cells or oocytes [56], which is compatible with the overall higher developmental competence observed in the medium-follicle group. At the pathway level, the significant enrichment of pyruvate metabolism in medium follicles suggests an optimized energy supply and redox balance conducive to maturation and subsequent cleavage [57,58]. Together, these metabolite- and pathway-level signatures align with the improved developmental outcomes observed for medium follicles in this study.

In contrast, although small follicles yielded the highest COC recovery rate (55.11%) and the highest proportion of top-quality oocytes (Grades A + B, 64.22%), their FF exhibits relatively reduced activity in energy metabolism and signaling pathways, including glycolysis [59,60], pyruvate metabolism [61], and CoA biosynthesis [62]. This pattern is in line with our observation that small follicles showed lower maturation (78.48%), cleavage (57.13%), and blastocyst (29.11%) rates than medium follicles, suggesting that limited metabolic support within the FF microenvironment may constrain meiotic progression and early embryonic development despite favorable recovery and morphology. For large follicles, we observed the lowest recovery rate (32.64%), the lowest proportion of Grades A + B oocytes (26.40%), and reduced developmental competence (maturation 71.28%; blastocyst 26.31%), accompanied by an oxidative FF profile (elevated MDA). Elevated MDA indicates enhanced lipid peroxidation and is consistent with the higher proportion of degenerated oocytes observed in the large-follicle group. Metabolomic analysis further revealed reduced hypoxanthine, which may lessen phosphodiesterase inhibition and decrease cAMP levels, thereby destabilizing meiotic arrest and promoting premature meiotic resumption [63,64], a mechanism consistent with the poorer competence of large-follicle oocytes in our IVP outcomes. In parallel, the upregulation of glutamine and anethole may represent an adaptive response to oxidative stress: glutamine can support glutathione synthesis and energy metabolism to improve IVM and fertilization [65], whereas anethole has been reported to modulate redox balance and mitochondrial function [66]. However, despite the increase in MDA, key antioxidant indicators (SOD, GPx, and T-AOC) did not show a compensatory rise, suggesting insufficient antioxidant buffering, which may allow oxidative damage to accumulate and ultimately impair mitochondrial integrity and oocyte developmental competence [67].

One limitation of this study is that follicle diameter was used as a practical surrogate for the underlying follicular environment, while oocyte developmental stage was not directly assessed and may change with follicle development. In addition, our COC grading was based only on cumulus investment and ooplasm appearance and does not indicate the nuclear maturation stage of the oocyte, which further limits interpretation. Therefore, we cannot fully separate the effect of follicle size per se from the effect of oocyte developmental stage. The differences observed among follicle size groups may thus reflect stage-related acquisition of competence and accompanying changes in the follicular microenvironment, rather than a direct causal effect of diameter itself. Future studies incorporating direct assessment of oocyte stage (e.g., GV status) and/or controlling follicular-wave stage will be needed to more clearly address size-specific mechanisms.

## 5. Conclusions

In conclusion, our results indicate that follicle size categories are significantly associated with oocyte developmental outcomes in high-producing dairy cows. Small follicles (2.0–5.9 mm) are a better source of high-quality oocytes, but the full realization of their developmental potential may require the optimization of in vitro maturation conditions. Oocytes from medium-sized follicles (6.0–9.9 mm) exhibited better developmental outcomes, including higher rates of maturation, cleavage and blastocysts, indicating that this cohort has the best “capability window” for the OPU-IVP protocol. In contrast, large follicles (≥10.0 mm) show signs of oocyte overmaturation and increased oxidative stress, thereby affecting the production efficiency of embryos. In assisted reproductive technology, excessive reliance on large follicle aspiration should be avoided. These findings provide empirical evidence for optimizing oocyte collection strategies and in vitro culture systems.

## Figures and Tables

**Table 1:** Recovery rate and mass distribution of COCs in small, medium and large follicles of dairy cows (LSM (95% CI)).

Item	Small Follicles (2.0–5.9 mm)	Medium Follicles (6.0–9.9 mm)	Large Follicles (10.0–20.0 mm)
Total follicles	1136	1009	383
Total COCs	626	471	125
follicles	10.42 (9.18–11.83) ^a^	9.26 (8.09–10.59) ^a^	3.51 (2.91–4.25) ^b^
COC recovery	5.74 (4.96–6.65) ^a^	4.32 (3.77–4.95) ^b^	1.15 (0.91–1.44) ^c^
COC recovery (%) ^1^	55.11 (51.85–58.32) ^a^	46.68 (44.23–49.15) ^b^	32.64 (29.39–36.06) ^c^
Grade A COCs	1.58 (1.22–2.04) ^a^	0.83 (0.65–1.05) ^b^	0.11 (0.05–0.22) ^c^
Grade B COCs	2.11 (1.80–2.47) ^a^	1.28 (1.03–1.57) ^b^	0.19 (0.12–0.31) ^c^
Grade C COCs	1.31 (1.08–1.59) ^a^	1.36 (1.11–1.67) ^a^	0.48 (0.34–0.66) ^b^
Grade D COCs	0.74 (0.56–0.99) ^a^	0.86 (0.64–1.16) ^a^	0.37 (0.24–0.56) ^b^
Grade AB COCs ^2^	3.69 (3.13–4.35) ^a^	2.10 (1.78–2.49) ^b^	0.30 (0.20–0.46) ^c^
COCs Grade distribution			
Grade A COCs (%)	27.48 (23.07–32.37) ^b^	19.11 (15.72–23.03) ^b^	9.60 (4.97–17.75) ^c^
Grade B COCs (%)	36.74 (32.80–40.86) ^a^	29.51 (24.52–35.05) ^b^	16.80 (10.82–25.16) ^c^
Grade C COCs (%)	22.84 (19.52–26.54) ^b^	31.42 (26.65–36.62) ^a^	41.60 (30.83–53.24) ^a^
Grade D COCs (%)	12.94 (10.21–16.27) ^c^	19.96 (15.52–25.28) ^b^	32.00 (22.26–43.61) ^a^
Grade AB COCs (%) ^3^	64.22 (60.06–68.18) ^a^	48.62 (43.29–53.98) ^b^	26.40 (18.68–35.90) ^c^
Cultured COCs	5.08 (4.39–5.89) ^a^	4.01 (3.46–4.65) ^b^	0.86 (0.67–1.11) ^c^
Cultured COCs (%) ^4^	88.01 (83.99–92.03) ^a^	86.28 (80.23–92.32) ^a^	75.89 (66.04–85.73) ^a^

Values are presented as least squares means (LSM) with 95% confidence intervals (CI). Models included follicle size (small, medium, and large) as a fixed effect and cow as a random effect. Proportional data were analyzed using generalized linear mixed models (GLMM), count data using negative binomial GLMM, and continuous data using linear mixed models (LMM). Pairwise comparisons were adjusted using the Holm method. Values within a row with different superscript letters (a–c) differ significantly (*p* < 0.05). ^1^ No. of total COC recovered/no. of total follicles. ^2^ No. of Grade A and Grade B COCs. ^3^ No. of Grade AB COCs/no. of total COCs. ^4^ No. of Cultured COCs/no. of COCs recovered.

**Table 2:** Developmental Capacity of COCs in Small, Medium and Large follicles of dairy cows and embryo Production (LSM (95% CI)).

Item	Small Follicles (2.0–5.9 mm)	Medium Follicles (6.0–9.9 mm)	Large Follicles (10.0–20.0 mm)
Mature oocytes	4.38 (3.83–5.02) ^a^	4.32 (3.78–4.93) ^a^	1.37 (1.16–1.61) ^b^
Cleaved oocytes	3.46 (2.94–4.08) ^a^	3.56 (3.07–4.12) ^a^	1.24 (1.02–1.52) ^b^
Blastocysts	1.63 (1.33–1.99) ^a^	2.01 (1.67–2.43) ^a^	0.59 (0.42–0.83) ^b^
Maturation (%) ^1^	78.48 (73.68–82.61) ^b^	89.93 (87.37–92.02) ^a^	71.28 (62.88–78.43) ^b^
Cleavage (%) ^2^	57.13 (51.64–62.63) ^b^	72.19 (67.83–76.55) ^a^	63.44 (52.80–74.07) ^a^
Blastocysts (%) ^3^	29.11 (25.19–33.38) ^b^	41.88 (37.51–46.37) ^a^	26.31 (17.91–35.79) ^b^

Values are presented as least squares means (LSM) with 95% confidence intervals (CI). Models included follicle size (small, medium, and large) as a fixed effect and cow as a random effect. Proportional data were analyzed using generalized linear mixed models (GLMM), count data using negative binomial GLMM, and continuous data using linear mixed models (LMM). Pairwise comparisons were adjusted using the Holm method. Values within a row with different superscript letters (a, b) differ significantly (*p* < 0.05). ^1^ No. of Mature oocytes/no. of Cultured COCs. ^2^ No. of cleaved oocytes/no. of Cultured COCs. ^3^ No. of blastocysts/no. of Cultured COCs.

## Data Availability

The original contributions presented in the study are included in the article/Supplementary Material, further inquiries can be directed to the corresponding authors.

## Supplementary Materials

The following supporting information can be downloaded at: https://www.mdpi.com/article/10.3390/ani16020274/s1, Table S1: Pooled raw counts (n/N) for oocyte recovery, COC grading, and in vitro developmental outcomes across follicle-size categories.
