# Supplementary material for: A Follicle Size Window of Competence for In Vitro Embryo Production in High-Producing Dairy Cows: Evidence from OPU-IVP Performance and Follicular Fluid Profiling
Source: Animals (Basel). 2026 Jan 16;16(2):274. doi: 10.3390/ani16020274 (PMC12837437; doi:10.3390/ani16020274)
Supplement: Supplementary file 1 [file animals-16-00274-s001.zip › animals-4071811-supplementary.pdf]

Supplementary Materials

Supplementary Table S1. Pooled raw counts (n/N) for oocyte recovery, COC grading, and in vitro developmental outcomes across follicle-size categories.

Item	Small follicles (2.0-5.9 mm)	Medium follicles (6.0-9.9 mm)	Large follicles (10.0-20.0 mm)
Aspirated follicles (n)	1136	1009	383
COC recovery, n (%)	626 (55.11%)	471 (46.68%)	125 (32.64%)
Grade A COCs, n (%)	172 (27.48%)	90 (19.11%)	12 (9.60%)
Grade B COCs, n (%)	230 (36.74%)	139 (29.51%)	21 (16.80%)
Grade C COCs, n (%)	143 (22.84%)	148 (31.42%)	52 (41.60%)
Grade D COCs, n (%)	81 (12.94%)	94 (19.96%)	40 (32.00%)
Grade AB COCs, n (%)[2]	402 (64.22%)	229 (48.62%)	33 (26.40%)
Cultured COCs, n (%)	554 (88.50%)	437 (92.78%)	94 (75.20%)
Mature oocytes, n (%)	431 (77.80%)	393 (89.93%)	67 (71.28%)
Cleaved oocytes, n (%)	343 (61.91%)	324 (74.14%)	61 (64.89%)
Blastocysts, n (%)	161 (29.06%)	180 (41.19%)	25 (26.60%)

Note: Values are shown as n (%), pooled across all cows for transparency. Statistical inference was conducted using cow-level mixed models (cow as the experimental unit); pooled counts are descriptive only.
